# Metallacarborane Derivatives Effective against *Pseudomonas aeruginosa* and *Yersinia enterocolitica*

**DOI:** 10.3390/ijms22136762

**Published:** 2021-06-23

**Authors:** Wieslaw Swietnicki, Waldemar Goldeman, Mateusz Psurski, Anna Nasulewicz-Goldeman, Anna Boguszewska-Czubara, Marek Drab, Jordan Sycz, Tomasz M. Goszczyński

**Affiliations:** 1 Laboratory of Medical Microbiology, Hirszfeld Institute of Immunology and Experimental Therapy, Polish Academy of Sciences, Weigla 12, 53-114 Wrocław, Poland; jordansycz@gmail.com; 2 Department of Organic and Medicinal Chemistry, Faculty of Chemistry, Wrocław University of Science and Technology, Wybrzeze Wyspianskiego 27, 50-370 Wrocław, Poland; waldemar.goldeman@pwr.edu.pl; 3 Laboratory of Experimental Anticancer Therapy, Hirszfeld Institute of Immunology and Experimental Therapy, Polish Academy of Sciences, Weigla 12, 53-114 Wrocław, Poland; mateusz.psurski@hirszfeld.pl (M.P.); anna.nasulewicz-goldeman@hirszfeld.pl (A.N.-G.); 4 Department of Medical Chemistry, Medical University of Lublin, 4A Chodzki Street, 20-093 Lublin, Poland; anna.boguszewska-czubara@umlub.pl; 5 USI, Unit of Nanostructural Biointeractions, Hirszfeld Institute of Immunology and Experimental Therapy, Polish Academy of Sciences, Weigla 12, 53-114 Wrocław, Poland; marek.drab@hirszfeld.pl; 6 Laboratory of Biomedical Chemistry, Hirszfeld Institute of Immunology and Experimental Therapy, Polish Academy of Sciences, Weigla 12, 53-114 Wrocław, Poland

**Keywords:** metallacarboranes, *Pseudomonas aeruginosa*, *Yersinia enterocolitica*, antibacterials, resistance generation, boron clusters, COSAN

## Abstract

*Pseudomonas aeruginosa* is an opportunistic human pathogen that has become a nosocomial health problem worldwide. The pathogen has multiple drug removal and virulence secretion systems, is resistant to many antibiotics, and there is no commercial vaccine against it. *Yersinia pestis* is a zoonotic pathogen that is on the Select Agents list. The bacterium is the deadliest pathogen known to humans and antibiotic-resistant strains are appearing naturally. There is no commercial vaccine against the pathogen, either. In the current work, novel compounds based on metallacarborane cage were studied on strains of *Pseudomonas aeruginosa* and a *Yersinia pestis* substitute, *Yersinia enterocolitica*. The representative compounds had IC_50_ values below 10 µM against *Y. enterocolitica* and values of 20–50 μM against *P. aeruginosa*. Artificial generation of compound-resistant *Y. enterocolitica* suggested a common mechanism for drug resistance, the first reported in the literature, and suggested *N*-linked metallacarboranes as impervious to cellular mechanisms of resistance generation. SEM analysis of the compound-resistant strains showed that the compounds had a predominantly bacteriostatic effect and blocked bacterial cell division in *Y. enterocolitica*. The compounds could be a starting point towards novel anti-*Yersinia* drugs and the strategy presented here proposes a mechanism to bypass any future drug resistance in bacteria.

## 1. Introduction

Resistance to current antibiotics is a problem gaining broad attention worldwide. The problem is considered extremely important by the World Health Organization and has led to the formation of several groups and initiatives aimed at finding a solution. The most notable of which is the CARB-X initiative based at Boston University, Boston, US, supported by the governments of the US, UK, and EU, as well as private foundations including the Bill and Melinda Gates foundation. The group prioritizes human pathogens of particular importance to public health which have been designated on the ESKAPE list [1,2]. Members of the group include *Pseudomonas aeruginosa*, a human pathogen for which there is no commercially available vaccine. The pathogen is known to have a high rate of antibiotic resistance acquisition and to be specifically troublesome in hospitals where close contact is responsible for the ease of transmission. Part of the problem may be the *Pseudomonas aeruginosa* genome that codes for multiple drug resistance pumps, posing challenges for antibiotic design [3]. The pathogen also possesses at least two major secretory systems: a type III secretion system (T3SS) [4], and the type VI secretion system (T6SS) [5]. The former is used mainly for defeating host immune systems, while the latter has a major role in defense against other bacteria [6] and fungi [7]. The T6SS also contributes to the defense of bacteria against the host, but its role is limited mainly to interference with inflammasome formation and signaling [8,9]. The type II secretion system (T2SS) [10]—a minor secretory system, as judged by its lesser role in infection—also contributes to the virulence of the pathogen.

Vaccine design against *Pseudomonas aeruginosa* has not reached a commercial stage despite many years of research and funding by different agencies and groups. Previous strategies used O-antigens as adjuvants but success was very low [11]. Inclusion of flagellins was attempted by different groups [12,13,14] but none were cleared commercially, despite at least one candidate reaching phase III clinical trials [15]. Porins were also trialed as vaccine candidates [16,17] but none were found to provide a benefit in human trials, despite promising results in animals [18,19]. Elements of the type III secretion system were also trialed as vaccine candidates. Inclusion of PcrV only in the vaccine was not sufficient to protect animal subjects completely based on survival rates [20,21,22,23,24,25]. However, immunization with rPcrV and subsequent challenge with a fully virulent strain administered with anti-PcrV antibodies could completely protect mice from a lethal dose of *P. aeruginosa* in a lung model of infection [26]. Other attempts used translocator proteins PopB/PopD [27,28] or the needle protein YscF [29]. In a change of strategy, outer membrane vesicles (OMVs) were used as vaccine candidates [30]. These constructs contain many secreted proteins and should be able to offer a polyvalent vaccine candidate. However, the survival rate was only 60–90% against different clinical strains.

Attempts to develop therapeutics targeting the offensive weaponry of the pathogen have faltered and also failed to reach the commercial stage [31,32,33,34,35]. Inhibition of other secretory systems, tat or T2SS, has been attempted with varying degrees of success [36,37]. None of these solutions, however, have passed all the human clinical trials.

*Yersinia pestis* is a zoonotic pathogen which infects humans. Historically, the pathogen is responsible for the Black Death epidemic in Europe which killed about a third of the population in the Middle Ages. The pathogen is the most lethal bacterium known to humans [38] and is of interest to the US and other countries’ militaries. It has resistance to many antibiotics, either engineered or natural [39,40,41], and represents a biosafety problem. These properties of pathogen, combined with the lack of commercial vaccines and emerg-ing vaccine candidate resistance in animals, add additional urgency to finding new ther-apeutics against *Yersinia pestis* and are of high importance for many countries to develop such countermeasures. Similar to *Pseudomonas aeruginosa*, the *Yersinia pestis* bacterium also codes for a specialized virulence factor delivery system, the T3SS, either chromosomally or on a plasmid [42]. The system is critical for the virulence of the pathogen [43] and its inactivation is a target of indirect-acting drugs including virulence blockers, which are developed by many groups [43,44]. Due to the structural similarity between many orthologous proteins from the T3SS, the drugs could offer a broad-spectrum specificity [43,45]. However, testing potential solutions against the pathogen requires high-security facilities which are costly and difficult to maintain. Therefore, substitute pathogens are used to test potential solutions. Common replacements are *Y. enterocolitica* [46] and attenuated strains of *Y. pestis* [47].

Existing antibiotics against *P. aeruginosa* and *Y. pestis* rely mainly on novel generations of β-lactamase and DNA gyrase/topoisomerase inhibitors [48,49,50]. Both classes have proven to be the only effective solutions after extensive use. The overreliance on these two classes led to a fast rise of resistance which could not be countered using traditional approaches. Therefore, new classes of antibiotics based on different strategies are needed and researched extensively. 

Modification of therapeutic scaffold by the inclusion of boron, mostly as a nucleophile trap, has been used in the past [51,52,53,54,55]. Other approaches include the addition of boron atoms or boron clusters linked to inhibitors, or metabolites used by bacteria [52,56,57,58,59]. This approach generated compounds with bacteriostatic properties and MIC values below 1 μM, and the antibacterial spectrum included *P. aeruginosa* and *S. aureus*—another member of the ESKAPE group.

In the era of newly emerging diseases and increasing problems of drug-resistant infections, there is an urgent need for the development of novel, unusual building blocks to design therapeutic molecules. Recently, there is an increasing interest in the biological and therapeutic activity of icosahedral boron cluster compounds, especially metallacarboranes [60,61,62]. Metallacarboranes are composed of two dicarbollide ligands *nido*-[C_2_B_9_H_11_]^2−^ that sandwich a central metal cation. The lead structure presented in this paper is metallacarborane containing the cobalt(III) ion—systematically named 3,3′-commo-bis[closo-1,2-dicarba-3-cobaltadodecaborate](1-) and often abbreviated as COSAN. The compound is highly stable under acidic and alkaline conditions, can easily penetrate cells, and is generally nontoxic to mammalian cells [63]. An 8-ethoxy-8′-iodo derivative of cobalt bis(1,2-dicarbollide) was shown as a novel antibacterial agent capable of fast-killing methicillin-resistant *Staphylococcus aureus* (MRSA) [64]. Other compounds based on the metallacarborane clusters were also shown to have antimicrobial and antibiofilm formation activities against different bacterial and fungal pathogens [63,65,66], including *Pseudomonas aeruginosa* and *Candida parapsylosis* [67].

Investigation of metallacarborane clusters as potential drug leads provided insight into their toxicity towards mammalian cells. COSAN, a cobaltabisdicarbollide anion, reversibly blocks cell growth in mammalian cell culture of most cells at concentrations above 100 µM. However, the addition of iodine atoms in I_2_-COSAN decreases the ED_50_ values approximately threefold [68]. The compounds, COSAN and I_2_-COSAN, could easily penetrate mammalian cells at lower concentrations and be removed over a longer period. Physical damage to cellular membranes was not observed and the effect on cells was only cytostatic.

Additionally, boron clusters are abiotic structures; living organisms lack enzymes that could metabolize these compounds. In contrast to organic molecules involved in life processes that have extensive hydrogen-bonding capabilities, boron clusters are incapable of forming classical hydrogen bonds and instead prefer dihydrogen bonds, additionally combining dispersed charge and hydrophobicity. The compounds are not recognized by cellular efflux systems, which is typically a major problem in anticancer therapy. Potential use against bacteria would be promising if the pathogens were not able to develop drug resistance.

In the current work, a new class of synthetic compounds based on COSAN and capable of blocking bacterial growth at low micromolar concentrations was identified. The compounds were evaluated as potential starting points for novel therapeutics against *P. aeruginosa* and a *Y. pestis* substitute, *Y. enterocolitica*. Artificial generation of resistance in *Y. enterocolitica* identified compounds capable of generating tolerance against the novel therapeutics. The data are in strong contrast with the reported results, where resistance to closely-related metallacarboranes could not be generated [64]. In the current manuscript, we generate resistance to the closely related compounds [64] and provide structural requirements necessary for avoiding drug resistance in metallacarboranes. It is the first report in the literature providing such information for this class of compounds.

## 2. Results

**Scheme 1:**
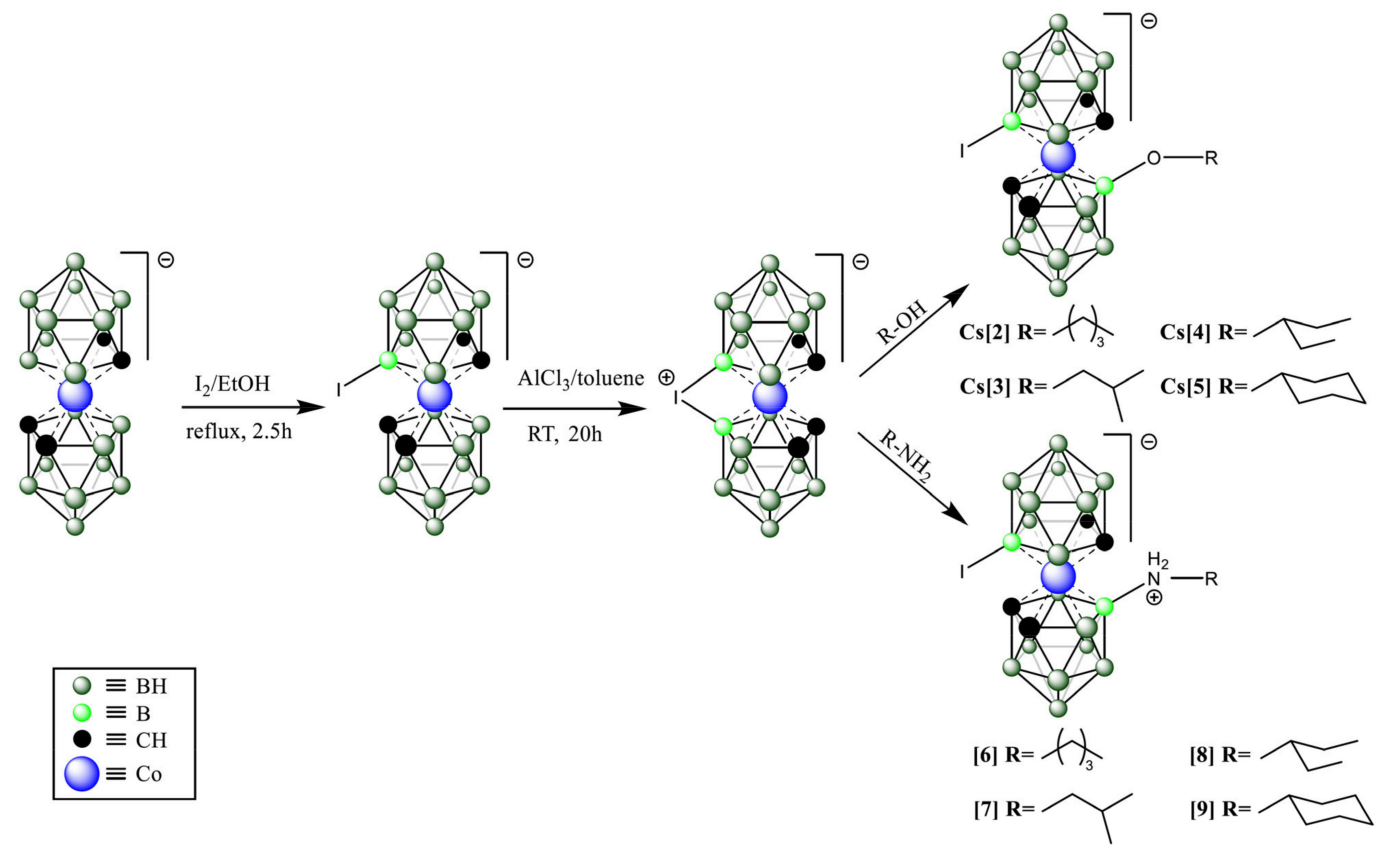
The synthetic scheme used to generate the top compounds from the screening strategy. Detailed synthetic procedures and analysis of compounds are provided under the Materials and Methods section.

For the synthesis of presented COSAN derivatives, we used iodonium bridge opening reaction (Scheme 1) with selected alcohols and amines [69,70,71]. We obtained an 8,8′-di-iodo derivative of COSAN (1) and two different groups of compounds with iodine atoms at the B(8′) position and a second organic substituent at B(8). Analogs were synthesized where the same organic part was linked via oxygen (B(8)-O bond) giving anionic products and the nitrogen atom (B(8)-N giving zwitterionic products.

### 2.1. Compound Screening and Selection

**Figure 1:**
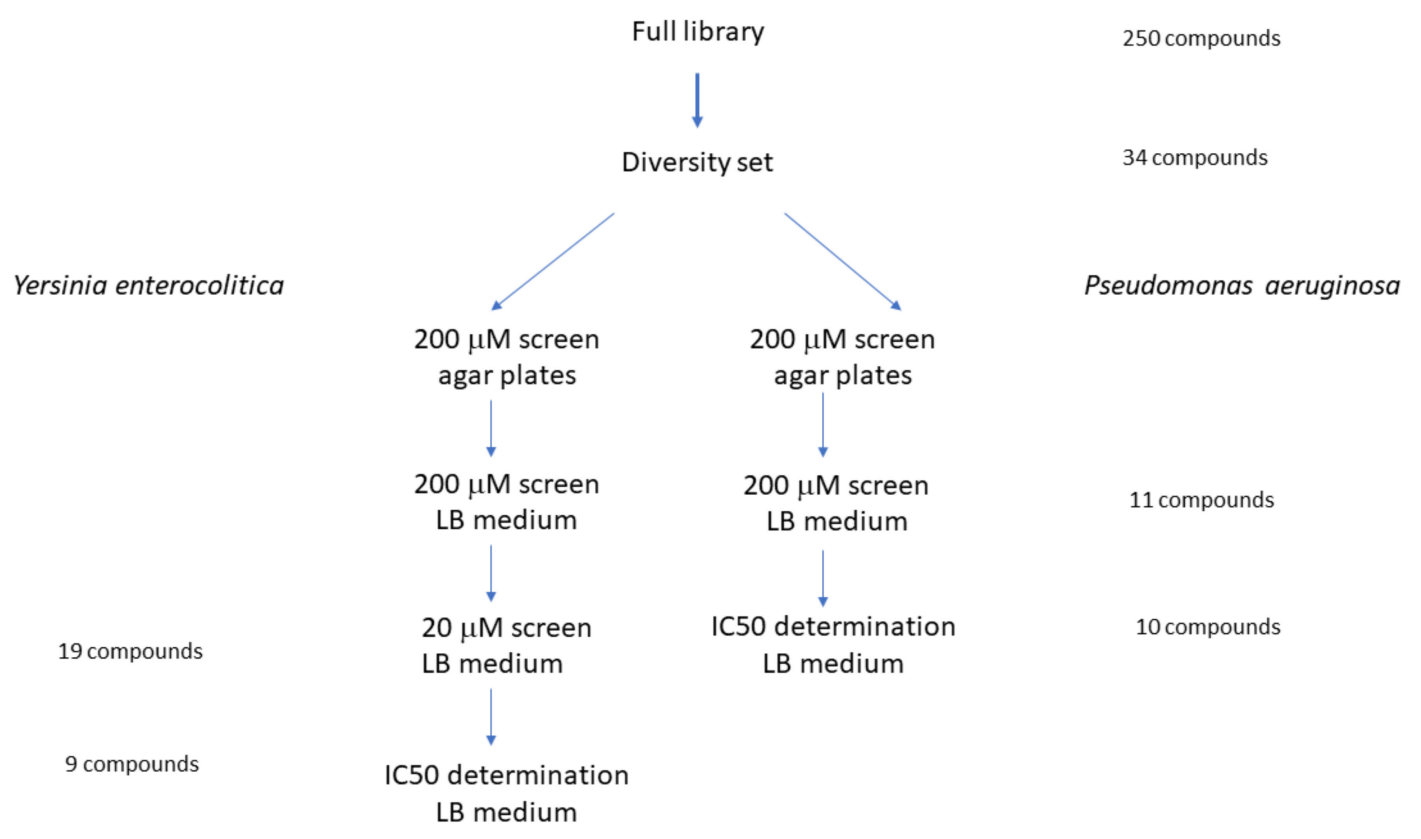
Screening strategy of small compounds library against *P. aeruginosa* and *Y. enterocolitica*. A diversity set of the complete library was screened on LB agar plates and then in a liquid medium as described under Materials and Methods.

Initial screening of small molecule metallacarborane library was performed in three stages (Figure 1).

**Figure 2:**
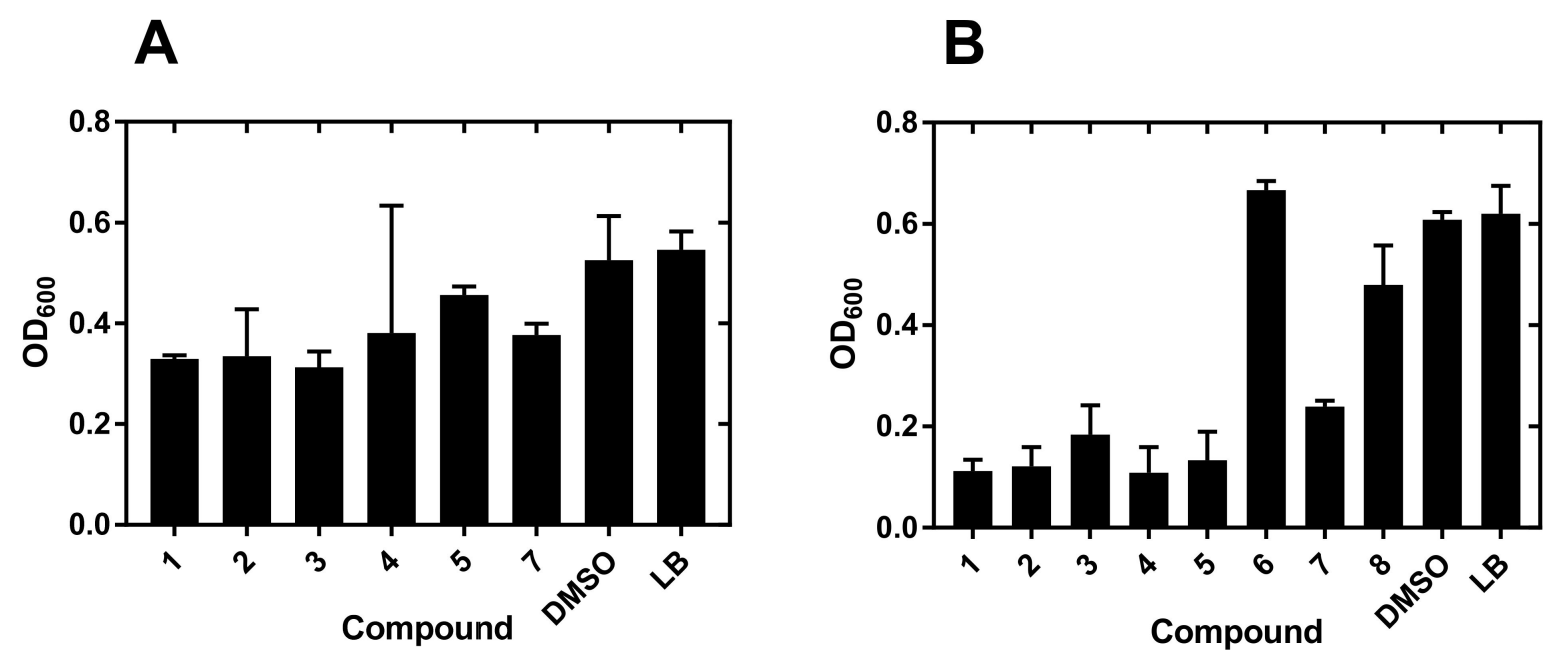
Growth inhibition of *Y. enterocolitica* by metallacarboranes. (**A**) Bacterial growth inhibitors selected from a 20 μM screen against *Y. enterocolitica*. Only compounds showing at least 50% growth inhibition are shown. (**B**) Bacteria were grown in 96-well flat-bottom plates at 37 °C in the presence of 200 µM compounds for 24 h. The growth was followed by measurement of OD600 absorbance as described under Materials and Methods. Measurements at 8 h post-inoculation are shown. Error bars correspond to the SEM value from 3–5 measurements. DMSO, dimethyl sulfoxide; LB, Luria-Bertani broth.

The diversity set used for the identification of potential hits selected compounds that had substantial antibacterial activity against *Yersinia enterocolitica* strain 2080 at 20 μM (Figure 2).

At this stage, the most active compounds were compounds 1–5 and 7, which were predominantly *O*-linked derivatives with two exceptions: the original compound 1 and the *N*-linked compound 7 (Scheme 1 and Table S1).

All compounds had short (four to five C atoms) aliphatic chains except for compound 5, which had a cyclohexyl ring.

Analogous strategy for the *Pseudomonas aeruginosa* strain 2058 did not produce significant hits at the 20 μM screening stage. The reason was a weak inhibition of growth which forced the selection of compounds from the 200 μM screening step (Figure 2).

The most active compounds were again 1–5 and 7, which were predominantly *O*-linked derivatives (Table S2) with two exceptions: the original compounds 1 and 7. 

Analysis of substitutions (Table S1 and Table S2) showed that the predominant part contained short (four to five C atoms) aliphatic chains. The exception was compound 5, with a cyclohexyl ring.

### 2.2. IC_50_ Determination

The candidate compounds were characterized further by the determination of IC_50_ values on two strains of each pathogen: *Y. enterocolitica* 2080 and 2090, and *P. aeruginosa* 2058 and 499 (Table 1). All measured IC_50_ values for *Y. enterocolitica* were below 10 μM and the trend was as observed for the 20 μM screen (Figure 2).

A literature search did not reveal any metallacarborane derivatives with anti-*Yersinia spp*. activities. However, boron-containing compounds were tested against *Yersinia ruckerii* and showed MIC values above 1500 μg/mL [72].

Compounds found in the current work are, then, the best growth inhibitors of *Yersinia spp*. reported in the literature. Structurally, they constitute a different class [72].

Activity against *Pseudomonas aeruginosa* was approximately an order of magnitude worse (Table S1) than for *Y. enterocolitica*. The trend in inhibition was as observed previously for the 200 μM screen (Table S2). Analysis of literature data indicates that the reported values are the best values measured for *Yersinia spp*. [73].

### 2.3. Metallacarborane Resistance Generation

Compounds found in the current work have unusual chemistry in addition to their antibacterial properties. Anionic boron clusters are man-made, inorganic compounds and as such living organisms lack enzymes that could metabolize them. It was, then, of interest to investigate if the compounds could be used to generate resistance against themselves and to elucidate a possible mechanism. To this end, *Y. enterocolitica* was selected as the pathogen of interest, based on the very low IC_50_ values. Three compounds were selected: 1, 7, and 3, showing different substitutions to the same scaffold. After at least nine cycles of growth, the compounds were tested against each other using a matrix-type approach (Table 2).

Only compound 7 could be defended against, regardless of the starting compound (Table 2, middle column). This result is startling as the almost identical compound 3 did not elicit the same resistance. At present, there is no explanation for the result.

### 2.4. SEM Investigation of Resistant Bacteria

The generation of cross-resistance to compound 7 suggests that the underlying mechanism of defense against COSAN derivatives may be common for all three compounds. Carborane clusters are not reported to be metabolized by any species. However, *nido*-carboranylporphyrins are easily uptaken by human, mouse, and rat cells and retained inside [74], possibly due to the lack of an efficient efflux system for these compounds.

**Figure 3:**
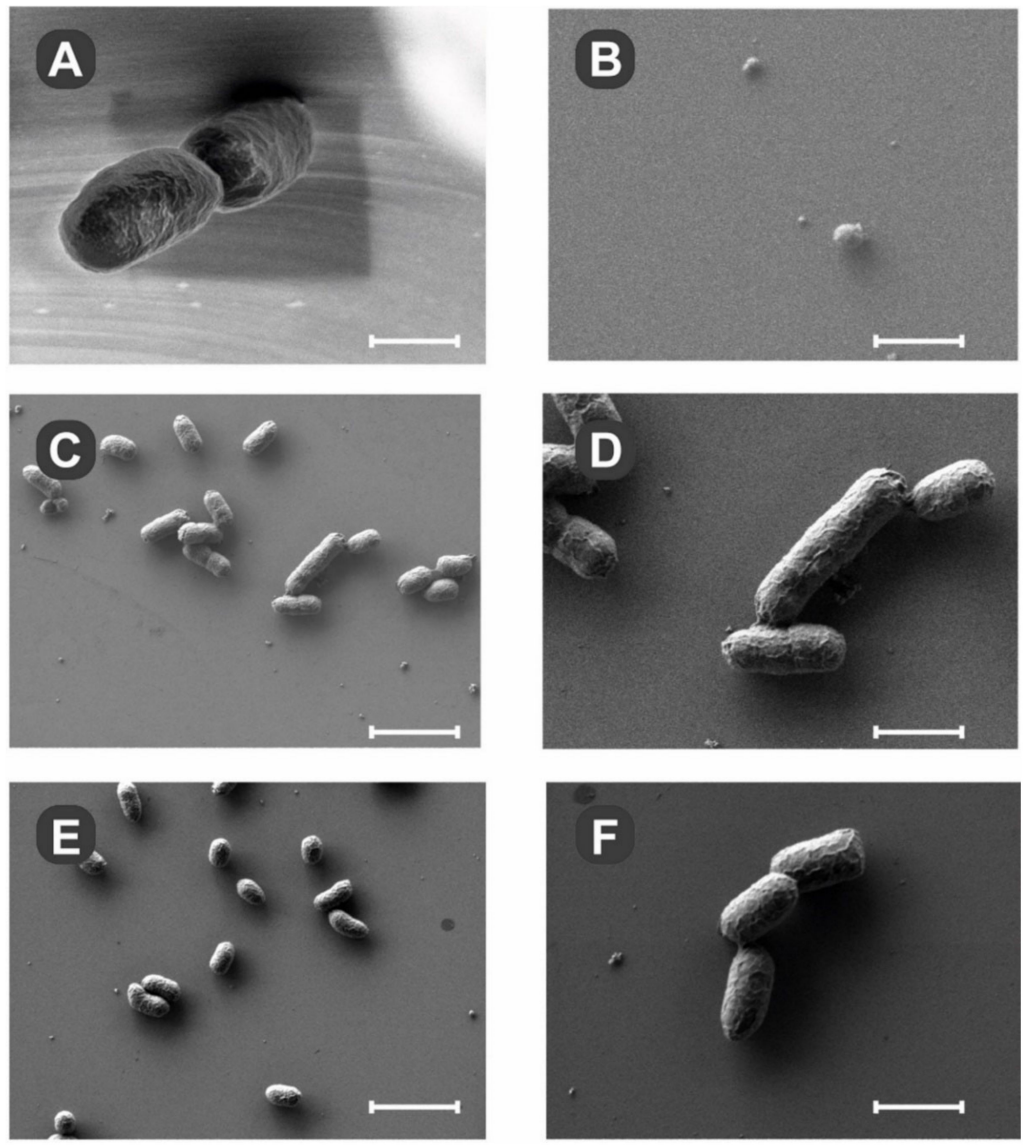
Scanning electron microscopy of drug-resistant *Y. enterocolitica* strain 2080. The SEM was performed on bacteria passaged at least nine times in the presence of 200 μM compound 1 (**B**), 7 (**C**,**D**), or 3 (**E**,**F**), and deposited on polished silicon chips as described in Materials and Methods. Bacteria grown without targeting compounds displayed normal morphology (**A**). After exposure to compound 1 for nine passages, there were only fragments of organic debris visible (**B**). In the presence of compounds 7 and 3, there were multiple bacteria observed with apparently normal morphology. Scale bars: (**A**) and (**B**) 500 nm, (**C**) and (**E**) 2.5 μm, (**D**) and (**F**) 1 μm.

To help us investigate the issue of potential mechanism, the drug-resistant variants of *Y. enterocolitica* 2080 strain were examined under SEM (Figure 3A–F).

**Figure 4:**
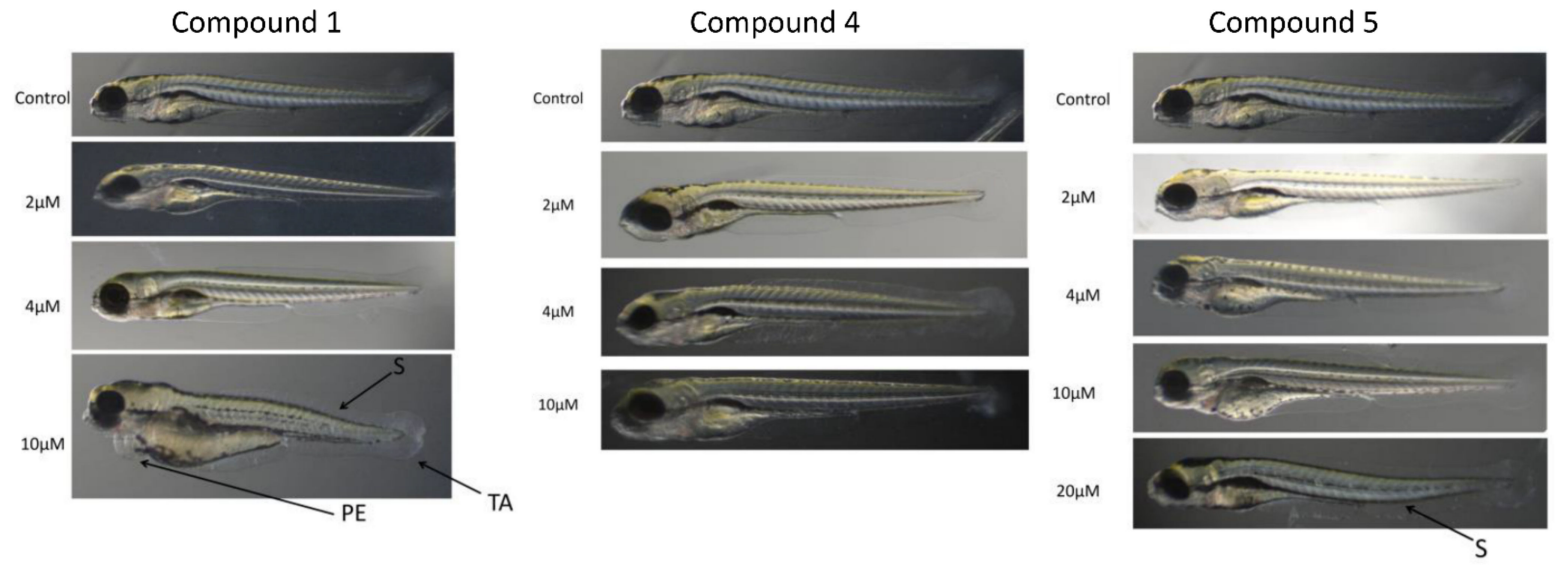
Toxicity of selected compounds on zebrafish (*Danio rerio*) larvae. Fish larvae were grown in 6-well plates in the presence of compounds for up to 5 days and the morphological deformities were analyzed from images as described under Materials and Methods. Representative photographs are shown. S, scoliosis; TA, tail autophagy; PE, pericardial edema.

Compound 1 generally inhibited bacterial growth and analysis of silicon chips showed only a few bacteria (Figure 3B) in the field of view. On the other hand, compounds 7 and 3 did not inhibit the growth of bacteria, and clusters of pathogens could be easily observed (Figure 4C,D and Figure 4E,F, respectively). The shapes of the bacteria treated with compounds 7 and 3 were comparable to the untreated ones. Morphologically, the cells looked very similar and maintained their integrity. The compounds’ mode of action seems to be bacteriostatic, targeting bacterial cell division.

### 2.5. Mammalian Cell Toxicity Studies

To measure potential toxicity towards mammalian cells, the compounds were tested for interference with cellular proliferation using two mammalian cell lines: the human lung epithelial cell line MCF 10A and mouse embryonic fibroblasts BALB/3T3 (Table 3, Figures S1 and S2).

For the MCF 10A cell line, the original compound 1 and the *O*-linked boron derivatives, 2–5, exhibited relatively low antiproliferative effects. In contrast, the *N*-linked boron derivatives, 6–9, showed high antiproliferative activity with IC_50_ values ranging from 40–160 nM. The results mirrored the data trend for the mouse BALB/3T3 line (Table 3). However, the IC_50_ values for the *O*-linked boron derivatives were about 4–5× lower than for the MCF 10A cell line. Compound 1 had an equal antiproliferative effect on both lines.

In summary, the antiproliferative effect was much smaller for the *O*-linked derivatives than for the *N*-linked ones. Results inversely correlated with the overall negative charge of the former, since its neutralization by the addition of a positive charge on the boron-linked nitrogen dramatically increased overall cellular toxicity.

### 2.6. Zebrafish Toxicity Studies

The final test for toxicity was performed on zebrafish. This animal is commonly used in toxicology studies.

The compounds chosen for animal studies were 1, 4, and 5. Compound 1 was shown to be an effective bacterial growth blocker for *Y. enterocolitica* (Table S1) and relatively nontoxic to mammalian cells (Table 3, Figures S1 and S2). Compound 4 was also a good bacterial growth blocker for *Y. enterocolitica* (Table S2) and was the least toxic compound towards the human epithelial cell line MCF 10A (Table 3 and Figure S1). Compound 5 was almost as effective as compound 4 towards *Y. enterocolitica* (Table S2) and was the second-best in terms of toxicity towards the human MCF 10A epithelial line (Table 3 and Figure S1).

In the zebrafish larvae toxicity studies (Figure 4), compound 1 induced visible morphological deformities in animals at 10 μM concentration, but not at the lower ones.

The changes were visible in the shape of the tail (TA), the dorsal string (S), and the heart (PE). Compounds 4 and 5 did not induce visible deformities at concentrations of up to 10 μM (Figure 4), the latter causing deformities of dorsal string only at 20 μM concentration.

**Figure 5:**
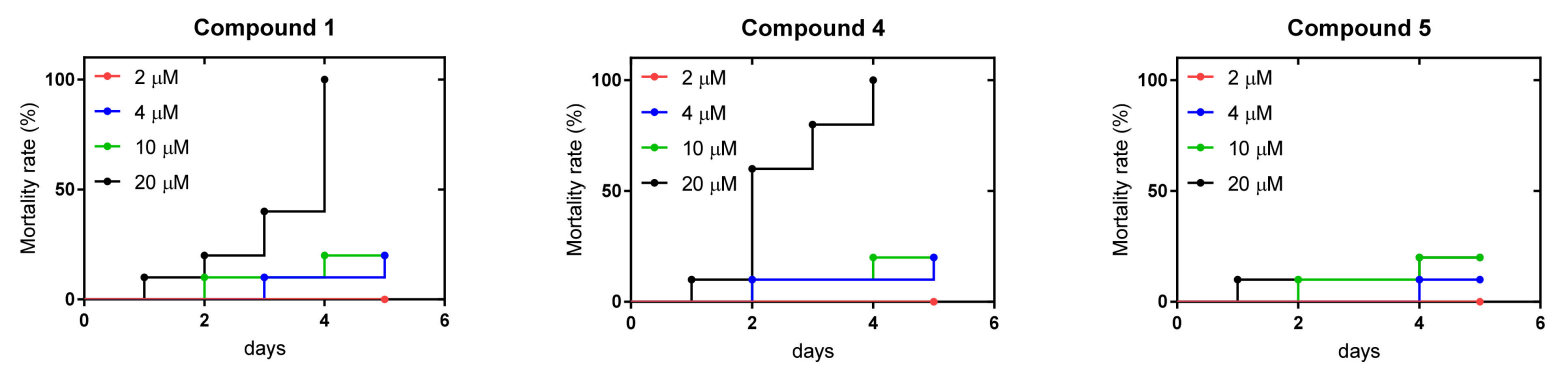
Mortality rates of selected compounds on zebrafish (*Danio rerio*) larvae. The population size (*n* = 10) from Figure 4 was followed up to day 5 as described under Materials and Methods. Representative graphs are shown.

Analysis of mortality rates for the compounds (Figure 5) showed that compound 5 had the lowest value of 20% at 20 μM concentration even at day 5 (Figure 5). 

Compounds 1 and 4 were lethal to all larvae on day 4 at the 20 μM concentration. At lower values, the mortality rates were only 20% even at day 5, except for the 2 μM concentration. In that case, none of the animals died up to day 5 post-exposure (Figure 5).

In summary, morphological deformities were not observed only for compounds 4 and 5 at the 10 μM concentration (Figure 4). The trend matched the mortality rates which showed that the concentration was not lethal to more than 20% of the population even at day 5 post-exposure. Compounds 4 and 5 were favored over compound 1 in the animal toxicity studies.

## 3. Discussion

Here we demonstrate new lead compounds based on boron-containing metallacarboranes and active against *Pseudomonas aeruginosa* and *Yersinia spp*. Our results indicate a novel mechanism for drug design that promises to overcome the drug resistance in these pathogens.

Widespread use of antibiotics and reliance on selected classes of drugs introduced to lower development costs have led to massive antibiotic resistance in human pathogens. This problem has been known for years but funding was not adequate and approval rates were too long to design new antibiotics before the emergence of massive resistance in the population. The situation is a public health problem for *Pseudomonas aeruginosa*, an ESKAPE-list pathogen. For *Yersinia pestis*, a class A select agent, the situation is not as critical due to the much better isolation procedures and active surveillance of potential outbreaks worldwide. In both cases, however, there is widespread resistance to antibiotics and a lack of commercial vaccines against either pathogen. In the current work, this problem was addressed by testing alternative scaffolds and chemistry as potential starting points to develop novel drugs suitable for these and other pathogens.

Metallacarboranes have extraordinary physicochemical properties and thus show encouraging biological activities. Amphiphilic behavior, interaction with proteins including inhibition of therapeutically important enzymes [75,76], low cytotoxicity towards mammalian cells [77], and, finally, promising perspectives for antimicrobial activity [78] make them very promising entities for designing drug candidates.

In the current work, we showed that a random screening of metallacarborane derivatives against two human pathogens, *Y. enterocolitica* as a substitute for *Y. pestis*, and *P. aeruginosa*, a member of the ESKAPE group, yielded compounds with biological activity against the former (Table S1) and the latter (Table S2). Expansion of testing against different strains of the two pathogens (Table 1) showed a good potency against *Y. enterocolitica* with IC_50_ values below 10 μM. The same compounds had approximately 5–10× higher IC_50_ values against *P. aeruginosa*, and the most biologically active compounds had a preference for short substitutions. At this stage, we do not have a good explanation for the preferences in substitutions or the overall lower biological activity of compounds against *P. aeruginosa* versus *Y. enterocolitica*.

Analysis of the literature for structures close to our compounds showed some similar metallacarborane scaffolds. Compound 1, also known as I_2_-COSAN, was shown as a good antibacterial agent against *K. pneumoniae* and *E. coli* [68]. The reported values of ED_50_ were at least twice and ten times as high as our reported values for *P. aeruginosa* and *Y. enterocolitica*, respectively (Table 1). 

The literature search also identified 8-ethoxy-8′-iodo derivative of cobalt bis(1,2-dicarbollide), compound K121 [64], as an analog of compound 2. The other compound was shown to have excellent antibacterial and antibiofilm formation properties against methicillin-resistant *S. aureus* (MRSA) [64].

*N*-linked derivatives of COSAN were tested against *P. aeruginosa* [67]. The closest analog in our work was compound 7 (Scheme 1). The IC_50_ values were about half the reported MIC_80_ values (Table 1 in [67]).

The introduction of longer chains connecting nucleophiles to the metallacarborane core was tested by another group [66]. The compounds differed from our derivatives by the lack of iodine attached to the metallacarborane cage. In their work, the compounds showed MIC_80_ values at least 2× higher than our values of IC_50_ measured against *P. aeruginosa* (Table 1). However, the other compounds were very effective against different fungi.

There have not been any reports showing the generation of resistance against metallacarborane derivatives. This phenomenon is very intriguing as conjugation with boron clusters is the basis of upcoming anticancer neutron therapy, and the generation of resistance would call into question many aspects of the strategy. Therefore, we tried to artificially generate resistance against three compounds: 1, 7, and 3 (Table 2). Surprisingly, only resistance to the *N*-substituted metallacarborane 7 could not be generated despite different starting points. The explanation may be the *N*-linkage in compound 7 as opposed to compound 1. However, it would not explain the ability to generate resistance to compound 1, which was the starting core.

The generation of resistance to metallacarboranes in our work is in strong contrast to the work by Zheng et al. [64]. In their work, the K121 metallacarborane was not able to generate resistance in MRSA after 20 passages. This difference may be attributed to a different mechanism of action, discussed later, and/or the selected pathogens and compounds used.

Metallacarboranes are man-made, extremely stable, caged structures; there are no reports in the literature showing biological degradation of this class of compounds. Removal of them was not shown, either, possibly due to the different recognition mechanisms by existing efflux systems. The compounds could potentially become the next generation of drugs if they had the proper characteristics.

To help us study the potential mechanism of biological activity, the drug-resistant pathogens were examined under SEM (Figure 3). Compound 1 simply blocked bacterial growth very efficiently and may have a bactericidal effect. Exposure to compounds 3 and 7 did not create visible cellular damage or changes in morphology in the drug-resistant variants. The mechanism of biological activity is most likely related to the blockage of bacterial cell division for compounds 3 and 7, with compound 1 most likely being bactericidal at the tested 200 μM concentration.

Work by Zheng et al. [64] showed that a metallacarborane K121, similar to our compound 2, most likely damaged bacterial membranes in *S. aureus* by the production of reactive oxygen species (ROS), a mechanism suggested for many nanoparticles [79,80,81]. In our study, the membranes of bacteria seemed to be intact as observed under SEM (Figure 3). This difference may be attributed to structural differences between the tested compounds and/or different strains of pathogens used in the studies. 

Assessment of toxicity for compounds was performed against two mammalian cell lines, the human non-tumorogenic lung epithelial MCF 10A cells, and mouse fibroblasts BALB/3T3. In both cases, the *O*-linked metallacarboranes showed lower toxicity than the *N*-linked ones. This trend may be caused by charge neutralization in *N*-linked compounds, as opposed to the negative charge in *O*-linked compounds.

Additional testing against zebrafish larvae was performed for compounds 1, 4, and 5 as the most promising candidates. At lower concentrations, all compounds were relatively safe for the larvae judging by the appearance of developmental deformities. However, at higher concentrations, it was clear that compounds 4 and 5 were superior to core compound 1 (Figure 4). This trend was confirmed by looking at the mortality rates (Figure 5).

Analysis of the available literature did not show the generation of resistance to metallacarboranes. In our studies, we showed that resistance can be generated to the core and *O*-linked compounds but not to the *N*-linked ones. The implication of this finding may have far-reaching consequences in cancer treatment where boron compounds are used to enhance the killing potential in neutron therapy. In the development of antibacterial compounds, this finding may form a basis for a novel antibacterial strategy where using *N*-linked metallacarboranes, as opposed to *O*-linked ones, is the key to avoiding future drug resistance.

## 4. Materials and Methods

### 4.1. Bacterial Strains and Media

Bacterial strains of *Yersinia enterocolitica* (PCM#2090, PCM#2080) were obtained from the Polish Collection of Microorganisms at the L. Hirszfeld Institute of Immunology and Experimental Therapy of Polish Academy of Science, Wroclaw, Poland. The *Pseudomonas aeruginosa* strain (PCM#2058) has a functional T3SS and was described previously [17]. Bacterial growth media (Luria-Bertani with agar) were prepared in-house using publicly available procedures. The blood agar plates were also prepared in-house using sheep blood and widely available procedures.

### 4.2. Cell Culture

BALB/3T3 (normal murine fibroblasts) and MCF 10A (human, non-tumorigenic mammary gland epithelial cells) were obtained from the American Type Culture Collection (ATCC; Rockville, Maryland, USA). The BALB/3T3 cell line was cultured in high glucose DMEM (Thermo Fisher Scientific) supplemented with 10% (*v*/*v*) fetal bovine serum (FBS) and 2 mM L-glutamine (Sigma-Aldrich). The MCF 10A cell line was cultured in Ham’s F12 medium with glutamine (Corning Costar) supplemented with 5% (*v*/*v*) FBS, 5% (*v*/*v*) horse serum, 10 µg/mL insulin, 0.05 µg/mL cholera toxin, 0.5 µg/mL hydrocortisone, and 20 ng/mL hEGF (all from Sigma-Aldrich). All culture media contained 100 µg/mL streptomycin (Sigma-Aldrich) and 100 U/mL penicillin (Polfa Tarchomin SA, Warszawa, Poland). The cells were grown at 37 °C in a humid atmosphere saturated with 5% CO_2_.

### 4.3. Antiproliferative Assay

Compounds’ antiproliferative activity was assessed utilizing the sulforhodamine B (SRB) assay [82] with minor modifications. Briefly, cells were seeded in 384-well plates (Greiner) at a density of 2 × 10^3^ cells/well, and after overnight incubation, the cells were exposed to compounds at a minimum of eight concentrations). After 72 h, the plates were fixed with 20% (*v*/*v*) TCA (Sigma-Aldrich), washed with tap water, and the precipitated proteins were labeled with 0.1% (*w*/*v*) SRB (Sigma-Aldrich) solution in 1% (*v*/*v*) AcOH. Next, the plates were washed with 1% (*v*/*v*) AcOH, and the remaining SRB dye was solubilized with 10 mM unbuffered TRIS (Sigma-Aldrich) solution. The entire procedure was performed using a semi-automatic washing station Biotek EL406 (BioTek Instruments, VT, USA). The absorbance was measured at 540 nm using a Biotek Hybrid H4 reader (BioTek Instruments, VT, USA). Mean proliferation inhibition was calculated for each compounds’ concentrations based on crude absorbance utilizing formula: %Inh = 100 − ((MeanAbsX − MeanAbsBLK)/(MeanAbsCTRL − MeanAbs-BLK)) × 100, where MeanAbsX is mean absorbance in compound/concentration-treated wells; MeanAbsCTRL is mean absorbance in vehicle-treated wells; and MeanAbsBLK is mean absorbance in cell-free wells. The results are expressed as the IC_50_ (compound concentration that reduced cell growth by 50%) calculated in GraphPad Prism 7.04 (GraphPad Software Inc., La Jolla, CA, USA) using the log(inhibitor_conc) vs. response (variable slope; four parameters) function.

### 4.4. *Danio rerio* Toxicity Assay

To determine acute toxicity of examined compounds modified Fish Embryo Toxicity (FET) test was performed of zebrafish (*Danio rerio*) according to OECD Test Guideline 236. Briefly, the collected embryos were transferred to a Petri dish with E3 medium (5 mM NaCl, 0.33 mM MgCl_2_, 0.33 mM CaCl_2_, 0.17 mM KCl; pH 7.2) and then placed in 24-well plates, 5 embryos per well, 10 per group. Stock solutions of boron cluster compounds were prepared in DMSO. In these experiments, three series of solutions with different concentrations were employed, which were freshly prepared by dissolving stock solutions in the E3 solution each time directly before addition to the wells. The solutions were changed once daily and the embryos were maintained in the incubator at 28.5 °C. Zebrafish embryos were evaluated for developmental abnormalities and viability at 24 h intervals for up to 5 days using a stereomicroscope (Zeiss Axio Vert, ZEISS, Germany). Morphological deformities of the heart, dorsal string, and tail development were measured and compared with the control embryos. Image analysis was performed to determine the percentage of dead and malformed embryos over time using Zeiss software. The experiment was performed in triplicate. Fish were taken from own fish facility of The Centre of Experimental Medicine, Medical University of Lublin. For the experiment AB OMD strain was used.

### 4.5. Chemical Reagents and Common Supplies

Cesium [3,3′-cobalt bis(1,2-dicarbollide)], Cs[COSAN] was purchased from Katchem (Prague, Czech Republic). DMSO (molecular biology grade, 99.9%+ purity) was purchased from Sigma-Aldrich (Sigma-Aldrich Sp. z o.o., Poznan, Poland). Premixed components for LB broth preparation were purchased either from Sigma-Aldrich (Sigma-Aldrich Sp. z o.o., Poznan, Poland) or from BioMaxima (BioMaxima SA, Lublin, Poland). Ninety-six-well sterile flat-bottom BioLite plates were purchased from Thermo Fisher Scientific (Thermo Fisher Scientific Polska Sp. z o.o., Warsaw, Poland). Solution basins and pipette tips were purchased from Sigma-Aldrich (Sigma-Aldrich Sp. z o.o., Poznan, Poland) and/or VWR (VWR International Sp. z o.o., Gdansk, Poland). Adhesive optically clear plate sealers were purchased from Sigma-Aldrich (Sigma-Aldrich Sp. z o.o., Poznan, Poland) and later from Thermo Fisher Scientific (Thermo Fisher Scientific Polska Sp. z o.o., Warszawa, Poland).

### 4.6. LC-MS Measurements

HPLC analyses were carried using the Ultimate 3000 RS HPLC system (Dionex, Sunnyvale, CA, USA) equipped with a DAD detector. High-resolution mass spectrometry experiments were carried out on a MicrOTOF-Q II spectrometer (Bruker Daltonic, Bremen, Germany) equipped with an electrospray ion source. The instrument was operated in the negative-ion mode and calibrated with a sodium formate solution (10 mM). The spectra were recorded using an acetonitrile/water mixture (80:20; *v*/*v*) containing 0.1% HCOOH.

### 4.7. NMR Spectroscopy

The ^1^H NMR, ^13^C{^1^H} NMR, and ^11^B{^1^H} NMR spectra were recorded on a 400 MHz Jeol ECZ 400S spectrometer in acetone-d_6_ (Sigma-Aldrich catalog number 151793) as a solvent in Wilmad quartz NMR tubes (Sigma-Aldrich catalog number Z562262) at 400 MHz, 100 MHz, and 128 MHz, respectively. Additionally, in selected cases DEPT135 and HMQC spectra were measured on the same spectrometer. Chemical shifts (δ) were expressed in parts per million (ppm), while multiplicity was reported as follows: s = singlet, d = doublet, t = triplet, q = quartet, quint = quintet, sext = sextet, hept = heptet, nonet = nonet, m = multiplet (complex pattern), br = broad. For clarity, the set of broad multiplets of BH protons at approximately 1–4 ppm was omitted in the ^1^H NMR description.

### 4.8. Chemical Synthesis

**Synthesis of Cs[8-I-3,3′-Co(1,2-C_2_B_9_H_10_)(1′,2′-C_2_B_9_H_11_)] (Substrate-A)** was performed according to literature methods [83] without any modification. Yield 86%.

**Synthesis of [8,8′-µ-I-3,3′-Co(1,2-C_2_B_9_H_10_)_2_] (Substrate-B)** was performed using the modification of previously described methods [69,70]. Briefly, AlCl_3_ (206 mg, 1.54 mmol, Sigma-Aldrich catalog number 294713) was added to a suspension of Cs[8-I-3,3′-Co(1,2-C_2_B_9_H_10_)(1′,2′-C_2_B_9_H_11_)] (Substrate-A): (300 mg, 0.515 mmol) in anhydrous toluene (70 mL) and was stirred for 20 h at room temperature under argon atmosphere. Then, the insoluble residue was filtered off and the filtrate was mixed with 0.4 g of silica gel for 5 min. The resulting solution was filtered and the solvent was evaporated. The red solid obtained was dried under vacuum for 24 h to give 220 mg (95%).

**Synthesis of Cs[8,8′-I_2_-3,3′-Co(1,2-C_2_B_9_H_10_)_2_] (1):** according to literature methods [84] without any modification. Yield 80%. ^1^H NMR (400 MHz, acetone-d_6_) δ: 4.38 (br s, 4 × BCH, 4H); ^11^B{^1^H} NMR (128 MHz, acetone-d_6_) δ: 1.86 (br s, 2B), −4.38 (br s, 8B), −6.34 (br s, 2B), −18.03 (br s, 4B), −23.56 (br s, 2B); ^13^C{^1^H} NMR (100MHz, acetone-d_6_) δ: 60.17 (br s, 4 × BCH). All spectra are in good agreement with literature data [84,85]. HPLC purity 98.8%, ESI-MS, [M]^-^ m/z (calcd/found) 576.0768/576.0781.

**General Method of Preparation of compounds 2–5**: approximately 100 mg of Substrate-B was dissolved in 10 mL of selected alcohol. The reaction mixture was left to stand for 20 h at room temperature (or in an oven thermostated at 40 °C, as indicated) under an argon atmosphere. The solvent was evaporated and a yellow solid was dried under vacuum. The crude product was dissolved in 8 mL of acetone and 300 mg CsCl in water (8 mL) was added. The resulting mixture was concentrated until the precipitation of an orange solid. This was filtered off (or centrifuged), washed with 3 mL of cold water and petroleum ether, and dried under a vacuum.

**Synthesis of Cs[8-O-*n*-Bu-8′-I-3,3′-Co(1,2-C_2_B_9_H_10_)_2_] (2):** 127 mg (0.28 mmol) of Substrate-B was dissolved in 10 mL anhydrous *n*-butanol (Sigma-Aldrich catalog number 281549). The reaction mixture was left to stand for 20 h at room temperature. Yield: 113 mg (61%). ^1^H NMR (400 MHz, acetone-d_6_) δ: 0.84 (t, CH_3_, 3H, *J* = 7.6 Hz), 1.27 (sext, CH_3_CH_2_, 2H, *J* = 7.6 Hz), 1.39 (quint, CH_3_CH_2_CH_2_, 2H, *J* = 7.4 Hz), 3.36 (br t, CH_2_O, 2H, *J* = 6.2 Hz); 4.18 (br s, 2 × BCH, 2H), 4.33 (br s, 2 × BCH, 2H); ^11^B{^1^H} NMR (128 MHz, acetone-d_6_) δ: 20.84 (br s, 1B), −1.50 (br s, 2B), −5.66 (br s, 2B), −6.66 (br s, 4B), −8.14 (br s, 3B), −18.93 (br s, 2B), −20.86 (br s, 2B), −24.34 (br s, 1B), −28.32 (s, 1B); ^13^C{^1^H} NMR (100 MHz, acetone-d_6_) δ: 13.34, 19.17, 33.96, 54.46 (br s, 2 × BCH), 56.45 (br s, 2 × BCH), 68.48. All spectra are in good agreement with literature data [70]. HPLC purity 95.3%, ESI-MS, [M]^−^ m/z (calcd/found) 522.2379/522.2395.

**Synthesis of Cs[8-O-*iso*-Bu-8′-I-3,3′-Co(1,2-C_2_B_9_H_10_)_2_] (3):** 130 mg (0.29 mmol) of Substrate-B was dissolved in 10 mL anhydrous *iso*-buthanol (Sigma-Aldrich catalog number 294829). The reaction mixture was left to stand for 20 h at room temperature. Yield: 109 mg (57.5%). ^1^H NMR (400 MHz, acetone-d_6_) δ: 0.78 (d, 2 × CH_3_, 6H, *J*= 6.8 Hz), 1.64 (nonet, CH, 1H, *J* = 6.8 Hz), 3.12 (d, CH_2_O, 2H, *J* = 6.4 Hz); 4.18 (br s, 2 × BCH, 2H), 4.37 (br s, 2 × BCH, 2H) ^11^B{^1^H} NMR (128 MHz, acetone-d_6_) δ: 20.75 (br s, 1B), −1.53 (br s, 2B), −5.63 (br s, 2B), −6.69 (br s, 4B), −8.25 (br s, 3B), −18.93 (br s, 2B), −20.84 (br s, 2B), −24.20 (br s, 1B), −28.43 (br s, 1B); ^13^C{^1^H} NMR (100MHz, acetone-d_6_) δ: 18.82, 30.19, 54.35 (br s, 2 × BCH), 56.47 (br s, 2 × BCH), 75.72. Note: on the ^1^H NMR spectrum additional signals of residual starting *iso*-butanol are present at 0.83 ppm and 3.26 ppm. The position of mentioned signals is in good agreement with literature data [86]. HPLC purity 95.0%, ESI-MS, [M]^-^ m/z (calcd/found) 522.2379/522.2370.

**Synthesis of Cs[8-O-CHEt_2_-8′-I-3,3′-Co(1,2-C_2_B_9_H_10_)_2_] (4):** 103 mg (0.23 mmol) of Substrate-B was dissolved in 10 mL anhydrous 3-pentanol (Sigma-Aldrich catalog number P8025). The reaction mixture was left to stand for 20 h at 40 °C. Yield: 118 mg (77%). ^1^H NMR (400 MHz, acetone-d_6_) δ: 0.76 (t, 2 × CH_3_, 6H, *J* = 7.6 Hz), 1.34 (br m, 2 × CH_3_CH_2_, 4H), 3.23 (br m, CHO, 1H,), 4.14 (br s, 2 × BCH, 2H), 4.36 (br s, 2 × BCH, 2H); ^11^B{^1^H} NMR (128 MHz, acetone-d_6_) δ: 20.61 (br s, 1B), −1.46 (br s, 1B), −2.25 (br s, 1B), −6.69 (br s, 6B), −8.32 (br s, 3B), −18.91 (br s, 2B), −20.63 (br s, 2B), −24.79 (br s, 1B), −28.75 (br s, 1B); ^13^C{^1^H} NMR (100 MHz, acetone-d_6_) δ: 9.14, 27.63, 55.13 (br s, 2 × BCH), 55.94 (br s, 2 × BCH), 79.31. HPLC purity 96.8%, ESI-MS, [M]^-^ m/z (calcd/found) 536.2537/536.2538.

**Synthesis of Cs[8-O-*cyclo*-C_6_H_11_-8′-I-3,3′-Co(1,2-C_2_B_9_H_10_)_2_] (5):** 98 mg (0.22 mmol) of Substrate-B was dissolved in 10 mL anhydrous cyclohexanol (Sigma-Aldrich catalog number 105899). The reaction mixture was left to stand for 20 h at 40 °C. Yield: 119 mg (80%). ^1^H NMR (400 MHz, acetone-d_6_) δ: 1.10–1.27 (m, CH_2_, 6H), 1.38 (m, CH_2_, 1H), 1.5–1.7 (m, CH_2_, 3H),~3.30 (br m, CHO, 1H)*, 4.17 (br s, 2 × BCH, 2H), 4.35 (br s, 2 × BCH, 2H); ^11^B{^1^H} NMR (128 MHz, acetone-d_6_) δ: 20.60 (br s, 1B), −1.68 (br s, 2B), −5.64 (br s, 2B), −6.68 (br s, 4B), −8.00 (br s, 3B), −18.95 (br s, 2B), −20.87 (br s, 2B), −24.46 (br s, 1B), −28.68 (br s, 1B); ^13^C{^1^H} NMR (100 MHz, acetone-d_6_) δ: 23.58, 25.74, 34.19, 55.12 (br s, 2 × BCH), 56.16 (br s, 2 × BCH), 75.14. Due to total overlap of the CH-O signal with signals of BH protons, its integration on the ^1^H NMR spectrum is overestimated. Its presence/position was confirmed by HMQC spectrum. HPLC purity 96.2%, ESI-MS, [M]^−^ m/z (calcd/found) 548.2537/548.2524.

**General Method of Preparation of compounds 6–9:** the selected amines were added to an approximately 100 mg suspension of Substrate-B in anhydrous cyclohexane (20 mL). The reaction mixture was stirred for 20–48 h at room temperature (or in an oven thermostated at 40 °C, as indicated) under an argon atmosphere. The desired compounds precipitated out of the reaction mixture as yellow solids. Then, the mixture was filtered and the solid was washed with cyclohexane and petroleum ether and dried under a vacuum.

**Synthesis of [8-NH_2_-*n*-Bu-8′-I-3,3′-Co(1,2-C_2_B_9_H_10_)_2_] (6):** 200 µL (2 mmol) of *n*-butylamine (Sigma-Aldrich catalog number 471305) was added to a 130 mg (0.29 mmol) suspension of Substrate-B in anhydrous cyclohexane (20 mL). The reaction mixture was stirred for 20 h at room temperature. The precipitated yellow product was filtered, washed with cyclohexane and petroleum ether, and dried under a vacuum. Yield: 128 mg, (84%). ^1^H NMR (400 MHz, acetone-d_6_) δ: 0.92 (t, CH_3_, 3H, *J* = 7.2 Hz), 1.43 (sext, CH_3_CH_2_, 2H, *J* = 7.2 Hz), 1.79 (m, CH_3_CH_2_CH_2_, 2H); 3.02 (br t, CH_2_N, 2H, *J* = 7.8 Hz), 4.42 (br s, 2 × BCH, 2H), 4.58 (br s, 2 × BCH, 2H), 6.80 (br s, 2H, NH_2_^+^); ^11^B{^1^H} NMR (128 MHz, acetone-d_6_) δ: 11.59 (br s, 1B), 2.10 (br s, 1B), −0.34 (br s, 1B), −2.43 (br s, 2B), −3.64 (br s, 1B), −4.75 (br s, 2B), −5.86 (br s, 2B), −7.74 (br s, 2B), −16.82 (br s, 2B), −17.75 (br s, 2B), −23.58 (br s, 2B); ^13^C{^1^H} NMR (100 MHz, acetone-d_6_) δ: 13.12, 19.71, 30.30, 49.46, 52.79 (br s, two overlapped signal all BCH carbons). HPLC purity 99.1%, ESI-MS, [M]^-^ m/z (calcd/found) 521.2539/521.2543.

**Synthesis of [8-NH_2_-*iso*-Bu-8′-I-3,3′-Co(1,2-C_2_B_9_H_10_)_2_] (7):** 100 µL (1 mmol) of *iso*-butylamine (Sigma-Aldrich catalog number I14150) was added to a 135 mg (0.30 mmol) suspension of Substrate-B in anhydrous cyclohexane (20 mL). The reaction mixture was stirred for 20 h in an oven thermostated at 40 °C. The precipitated yellow product was filtered, washed with cyclohexane and petroleum ether, and dried under a vacuum. Yield: 146 mg, (93%). ^1^H NMR (400 MHz, acetone-d_6_) δ: 1.07 (d, 2 × CH_3_, 6H, *J* = 6.8 Hz), 2.15 (nonet, (CH_3_)_2_CH, 1H, *J* = 7.2 Hz), 2.95 (m, CH_2_N, 2H), 4.43 (br s, 2 × BCH, 2H), 4.58 (s, 2 × BCH, 2H), 6.71 (br s, 2H, NH_2_^+^); ^11^B{^1^H} NMR (128 MHz, acetone-d_6_) δ: 11.58 (br s, 1B), 2.13 (br s, 1B), −0.58 (br s, 1B), −2.34 (br s, 2B), −3.76 (br s, 1B), −4.72 (br s, 2B), −5.88 (br s, 2B), −7.58 (br s, 2B), −16.78 (br s, 2B), −17.70 (br s, 2B), −23.67 (br s, 2B); ^13^C{^1^H} NMR (100 MHz, acetone-d_6_) δ: 19.80, 27.76, 52.69 (br s, 2 × BCH), 52.82 (br s, 2 × BCH), 56.60. HPLC purity 96.6%, ESI-MS, [M]^-^ m/z (calcd/found) 521.2539/521.2516.

**Synthesis of [8-NH_2_-CHEt_2_-8′-I-3,3′-Co(1,2-C_2_B_9_H_10_)_2_] (8**)**:** 200 µL (1.7 mmol) of 3-aminopentane (Acros catalog number 183700250) was added to a 110 mg (0.25 mmol) suspension of Substrate-B in anhydrous cyclohexane (20 mL). The reaction mixture was stirred for 48 h at room temperature. The precipitated yellow product was filtered, washed with cyclohexane and petroleum ether, and dried under a vacuum. Yield: 121 mg, (92%). ^1^H NMR (400 MHz, acetone-d_6_) δ: 0.99 (t, 2 × CH_3_, 6H, *J* = 7.6 Hz), 1.86 (m, 2 × CH_3_CH_2_, 4H), 3.07 (br m, CHN, 1H,), 4.49 (br s, 2 × BCH, 2H), 4.65 (s, 2 × BCH, 2H), 6.19 (br s, 2H, NH_2_^+^); ^11^B{^1^H} NMR (128 MHz, acetone-d_6_) δ: 11.21 (br s, 1B), 2.21 (br s, 1B), −0.18 (br s, 1B), −2.56 (br s, 2B), −3.98 (br s, 1B), −4.74 (br s, 2B), −5.56 (br s, 2B), −7.74 (br s, 2B), −16.73 (br s, 2B), −17.91 (br s, 2B), −23.65 (br s, 2B); ^13^C{^1^H} NMR (100 MHz, acetone-d_6_) δ: 9.07, 24.29, 52.64 (br s, 2 × BCH), 53.71 (br s, 2 × BCH), 63.53. Note: on ^1^H NMR and ^13^C{^1^H} NMR spectra a peak of residual cyclohexane (reaction solvent) is present at 1.39 ppm and 26.68 ppm, respectively. The position of mentioned signals is in good agreement with literature data [87]. HPLC purity 99.6%, ESI-MS, [M]^-^ m/z (calcd/found) 535.2696/535.2691.

**Synthesis of [8-NH_2_-*cyclo*-C_6_H_11_-8′-I-3,3′-Co(1,2-C_2_B_9_H_10_)_2_] (9):** 80 µL (0.7 mmol) of cyclohexylamine (Sigma-Aldrich catalog number 240648) was added to a 150 mg (0.33 mmol) suspension of Substrate-B in anhydrous cyclohexane (20 mL). The reaction mixture was stirred for 48 h at room temperature. The precipitated yellow product was filtered, washed with cyclohexane and petroleum ether, and dried under a vacuum. Yield: 144 mg, (78%). ^1^H NMR (400 MHz, acetone-d_6_) δ: 1.18 (m, CHH, 1H), 1.32 (m, CHH + CHH, 2H), 1.55 (m, CHH+CHH, 2H), 1.62 (m, CHH, 1H), 1.78 (m, 2 × CHH, 2H), 2.30 (m, 2 × CHH, 2H), 3.11 (br tt, CHN, 1H, J = 11.2 Hz, J = not resolve), 4.47 (br s, 2 × BCH, 2H), 4.62 (s, 2 × BCH, 2H), 6.41 (br s, 2H, NH_2_^+^); ^11^B{^1^H} NMR (128 MHz, acetone-d_6_) δ: 11.23 (br s, 1B), 2.07 (br s, 1B), −0.34 (br s, 1B), −2.62 (br s, 2B), −3.79 (br s, 1B), −4.82 (br s, 2B), −5.65 (br s, 2B), −7.59 (br s, 2B), −16.76 (br s, 2B), −17.82 (br s, 2B), −23.62 (br s, 2B); ^13^C{^1^H} NMR (100 MHz, acetone-d_6_) δ: 24.57, 24.86, 31.77, 52.58 (br s, 2 × BCH), 53.80 (br s, 2 × BCH), 59.86. Note: as in the case of compound 8, on ^1^H NMR and ^13^C{^1^H} NMR spectra a peak of residual cyclohexane (reaction solvent) is present at 1.39 ppm and 26.68 ppm, respectively. HPLC purity 98.0%, ESI-MS, [M]^-^ m/z (calcd/found) 547.2697/547.2700.

### 4.9. Library Screening for Bacteria Growth Inhibition

A fresh LB agar plate was warmed up to RT for about 30 min and a 200 μL aliquot of 1:100 dilution of a fresh overnight culture of bacterial stock grown in 3 mL of LB medium was spread on the plate. The plate was left to dry under a biological safety cabinet (BSC) for approximately 30 min and a 10 μL aliquot of the compound in PBS at 200 μM concentration was added to the plate. Bacteria with the compound added were left under the BSC for another 30 min to dry before the plates were covered with a lid and put in a 37 °C incubator. The next day, plates were scored visually for bacterial growth inhibition: (a) strong—for a pronounced halo (5–8 mm diameter); (b) weak—for a visible disturbance of uniform bacterial growth at the site of compound application; and (c) no inhibition- for a lack of visible disturbance of bacterial lawn.

### 4.10. Bacterial Growth Inhibition IC_50_ Measurements

Bacterial growth inhibition was measured on a Spark 10 M (Tecan, AT) microplate reader as described previously (Swietnicki et al., 2019). Briefly, a bacterial culture in LB medium grown at 37 °C overnight was diluted 1:100 with a fresh medium and aliquoted into a 96-well plate at 98 μL/well with a multichannel pipette. A 2 μL aliquot of compound serially diluted in DMSO was added in triplicates, the plate was covered with a lid or a transparent plate sealer, and incubated at 37 °C for up to 24 h. Bacterial growth was monitored by following an A_600_ reading every hour. After the experiment, the data were stored in Excel format, transferred into KaleidaGraph software (Synergy Software, Reading, PA, USA) and the A_600_ was plotted versus log [Concentration]. The IC_50_ values were determined by fitting the readings at the 8 h time point to a sigmoidal model of inhibition. Each experiment was repeated at least 3 times to verify the values.

### 4.11. Compound Resistance Evolution

The evolution was performed in parallel using blood agar and LB agar plates prepared in-house. Briefly, a 200 μL aliquot of 200 μM compound in PBS was added to a fresh agar plate, spread on the surface, and left to dry in a BSC cabinet for approximately 30 min. After that time, a single colony from an overnight culture on a plate was spread on the surface before the plate was covered with a lid and left at 37 °C to incubate overnight. The procedure was repeated at least nine times for each plate. After the last step, a single colony was selected from a plate and added to a 3 mL aliquot of LB medium. The culture was incubated at 37 °C overnight and used later as a stock for the IC_50_ determination of growth inhibition by compounds, as described previously. Dual selection on different plates was necessary in cases where the growth inhibition was too strong to obtain single colonies on LB agar plates. Typically, the colonies showed up on either medium after the third to fourth passage. Until then, the colonies were supplied from bacteria grown on the blood agar plates.

### 4.12. Scanning Electron Microscopy (SEM)

Scanning electron microscopy of *Y. enterocolitica* was performed at a low accelerating voltage of the primary beam without any coating or heavy-metal contrasting of the samples, as described in previous work [88]. Bacteria were applied onto a polished silicon crystal chip and allowed to adhere for 30 min. The samples were fixed with 2.5% glutaraldehyde in 0.1 M cacodylate buffer for 30 min at 4 °C, then washed in water and dehydrated in a series of methanol solutions (25%-50%-75%-100%-100%) in one-hour steps at 4 °C. Samples underwent critical point drying with methanol exchanged for liquid CO_2_ in an automatized approach, (CPD300 AUTO, Leica Microsystems, Vienna, Austria) and imaged with a cross-beam scanning electron microscope equipped with a Schottky field-emission cathode (Auriga 60, Carl Zeiss, Oberkochen, Germany) at 0.8 kV accelerating voltage. Thus, the imaging was performed within a mode referred to as the low-voltage, field-emission scanning electron microscopy (LV-FESEM) of nonlabeled, critical point-dried samples.

## Figures and Tables

**Table 1:** Top hits IC_50_ values for growth inhibition of selected pathogens.

Compound No.	Pathogen			
	Ye 2080 ^a^	Ye 2090 ^b^	Pa 2058 ^c^	Pa499 ^d^
**1**	4.8 ± 3.0	2.5 ± 1.4	28.3 ± 6	11.4 ± 1
**2**	4.3 ± 1.5	2.4 ± 0.7	28 ± 8	12.1 ± 4
**3**	4.2 ± 1.8	3.4 ± 1.0	59 ± 30	16.9 ± 8
**4**	6.7 ± 2.3	5.3 ± 1	72 ± 30	52.9 ± 20
**5**	7.5 ± 4	3.3 ± 1	52.1 ± 15	52.1 ± 10
**7**	1.8 ± 0.8	6.2 ± 0.7	24.7 ± 8	58.7 ± 20

IC_50_ determination was performed at the 8-h time point of growth in Luria-Bertani medium at 37 °C as described under Materials and Methods. Measurements were performed in triplicate and the errors refer to SEM from three to five independent experiments. ^a^ *Ye 2080*, *Yersinia enterocolitica* strain PCM 2080; ^b^ *Ye 2090*, *Yersinia enterocolitica* strain PCM 2090; ^c^ *Pa 2058, Pseudomonas aeruginosa* strain PCM 2058; ^d^ *Pa 499, Pseudomonas aeruginosa* strain PCM 499.

**Table 2:** Cross-resistance generation in *Y. enterocolitica* for selected compounds.

Compound No.	Resistance ^a^		
	1	3	7
**1**	2.6 ± 0.2	4.7 ± 1	>37
**3**	4.0 ± 1	10.4 ± 3	>100
**7**	3.1 ± 1	3.7 ± 1.2	>100

*Y. enterocolitica* strain PCM 2080 was grown in the presence of 200 μM compounds on LB agar plates at 37 °C for at least nine generations. A single colony was used for IC_50_ determination in LB medium as described under Materials and Methods. ^a^, Resistance was determined as an IC_50_ value against the selected compound.

**Table 3:** Antiproliferative activity of the tested compounds assessed by the SRB method after 72 h of treatment in two diverse cell lines. The highest concentration used was 100 μM.

Compound No.	$IC_{50}^{72h}\;(95\%\;\mathrm{CI})$	
	**BALB/3T3**	**MCF 10A**
**1**	17.0 (11.4–36.9)	59.8 (ND)
**2**	3.7 (2.3–6.7)	16.1 (9.08–239)
**3**	10.2 (8.2–13.3)	52.5 (30–63)
**4**	11.3 (9.9–14.0)	50 (ND)
**5**	11.7 (9.3–15.2)	46.3 (20.0–54.4)
**6**	0.29 (0.17–0.57)	0,16 (0.098–0,28)
**7**	0.024 (0.017–0.034)	0.115 (0.084–0.16)
**8**	0.023 (0.016–0.033)	0.045 (0.041–0.050)
**9**	0.067 (0.043–0.11)	0.082 (0.068–0.100)

## Data Availability

The datasets used and/or analyzed during the current study are available from the corresponding authors on reasonable request.

## Supplementary Materials

The following are available online at http://doi.org/10.5281/zenodo.4776055, compounds characterization, supplementary figures—Figures S1 and S2; Supplementary tables—Tables S1 and S2.
